# Development of Photoremovable Linkers as a Novel Strategy to Improve the Pharmacokinetics of Drug Conjugates and Their Potential Application in Antibody–Drug Conjugates for Cancer Therapy

**DOI:** 10.3390/ph15060655

**Published:** 2022-05-25

**Authors:** Audrey Nathania Johan, Yi Li

**Affiliations:** 1 Department of Chemistry, Xi’an Jiaotong-Liverpool University, Suzhou 215123, China; audrey.johan17@student.xjtlu.edu.cn; 2 Academy of Pharmacy, Xi’an Jiaotong-Liverpool University, Suzhou 215123, China

**Keywords:** photoremovable linker, drug conjugates, antibody–drug conjugates

## Abstract

Although there have been extensive research and progress on the discovery of anticancer drug over the years, the application of these drugs as stand-alone therapy has been limited by their off-target toxicities, poor pharmacokinetic properties, and low therapeutic index. Targeted drug delivery, especially drug conjugate, has been recognized as a technology that can bring forth a new generation of therapeutics with improved efficacy and reduced side effects for cancer treatment. The linker in a drug conjugate is of essential importance because it impacts the circulation time of the conjugate and the release of the drug for full activity at the target site. Recently, the light-triggered linker has attracted a lot of attention due to its spatiotemporal controllability and attractive prospects of improving the overall pharmacokinetics of the conjugate. In this paper, the latest developments of UV- and IR-triggered linkers and their application and potential in drug conjugate development are reviewed. Some of the most-well-researched photoresponsive structural moieties, such as UV-triggered coumarin, ortho-nitrobenzyl group (ONB), thioacetal ortho-nitrobenzaldehyde (TNB), photocaged C40-oxidized abasic site (PC4AP), and IR-triggered cyanine and BODIPY, are included for discussion. These photoremovable linkers show better physical and chemical stabilities and can undergo rapid cleavage upon irradiation. Very importantly, the drug conjugates containing these linkers exhibit reduced off-target toxicity and overall better pharmacokinetic properties. The progress on photoactive antibody–drug conjugates, such as antibody–drug conjugates (ADC) and antibody–photoabsorber conjugate (APC), as precision medicine in clinical cancer treatment is highlighted.

## 1. Introduction

More than 100 years have passed since the first concept of chemotherapy and magic bullet were introduced by Paul Ehrlich [1]. Since then, much research and development have been made to advance the concept, yet cancer still remains one of the major leading causes of death, accounting for approximately 10 million deaths worldwide, with around 19.3 million new cases in 2020 [2]. Since the approval of nitrogen mustard Mustine by the FDA in 1949 as a chemotherapy agent, many cytotoxic agents have been discovered [3]. In general, these cytotoxic agents can be categorized into antifolate (methotrexate); potent tubulin inhibitors such as amanitins, maytansinoid, auristatin, vinca alkaloids, and taxanes; and DNA damaging agents such as anthracyclines (doxorubicin), enedyines (calicheamicin, neocarzinostatin, and esperamicins), cyclopropaindoles (duocarmycin and CC-1065), indolobenzodiazepines, pyrrolobenzodiazepines, as well as several other types as well [4,5].

Unfortunately, the further development of these drugs is proven to be difficult due to the poor pharmacokinetic properties along with the safety concerns related to systemic toxicity and lack of targeting agents. Therefore, many approaches have been adopted to improve the pharmacokinetics of the drug, the pharmacokinetic properties such as solubility, permeability, systemic stability/circulatory half-life, selective toxicity, extravasation rates, and retention time in the tumor microenvironment remain to be the main foci of these efforts. 

**Figure 1:**
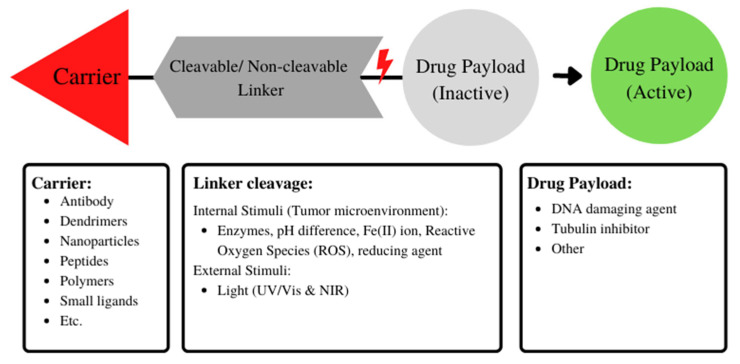
Schematic representation of various drug conjugates.

Among these approaches, drug conjugates have become one of the interesting strategies to be implemented. The drug is attached to the carrier by a linker through covalent conjugation to render it inactive during circulation (Figure 1). Then, the drug is released through decomposition of the linker by either external or internal stimuli after reaching the tumor microenvironment. 

As seen in Figure 1, the carrier can have significant influences on the pharmacokinetic properties and may act as a targeting agent to reduce the off-target toxicity of the drug conjugates. There are many substrates that are eligible to be the carriers of the drug, for example, monoclonal antibodies (mAb), dendrimers, small ligands, polymers, nanoparticles, peptides, and many more [6]. On the other hand, the linker plays a determinant role in the drug conjugate cleavage, as well as affecting the stability, hydrophilicity, absorption, distribution, metabolism, and excretion (ADME) properties of the overall drug molecule [7].

There are two types of linkers developed so far, different in where the stimuli for the cleavage triggering mechanism are from. The internal trigger of the first type of linker takes advantage of the difference in systemic conditions between the normal tissue and the tumor microenvironment. For example, an acid-sensitive hydrazone linker becomes cleavable in the tumor tissue because of the lower pH condition, and enzyme cleavable, dipeptide, and disulfide linkers are susceptible to the overpresence of trigger enzymes such as cathepsin and glutathione (GSH) in the tumor environment. Although extensive progress has been made on internal trigger strategy, moderate stability and off-target release of the drug still restrict their therapeutic index due to the non-specific presence of the trigger in the non-target environment. Additionally, varying chemical environments between patients and lack of artificial control post-administration also become one of the main problems of this linker strategy. Therefore, there is an emerging interest in utilizing external triggers in drug delivery systems. Some of the external triggers already implemented in drug delivery systems include light, radiation, heat, magnetic field, ultrasound, electric, chemical “click and release” trigger, etc. [8,9,10,11,12]. Apart from the improved stability of the drug conjugate, some extra advantages of these external stimuli, such as more precise controls over timing, location, and dosage, allow for tailoring the drug delivery in different cases. 

Among all the external stimuli mentioned, light gains a special status due to its non-invasive approach and long history of successful application in therapy. Its clean and easily obtainable simulation is highly efficient and manipulatable with highly spatiotemporal precision [13,14,15]. One of the well-known applications of light trigger in clinical practice is photodynamic therapy (PDT), where photosensitizers upon irradiation produce singlet oxygen, the highly reactive radical to induce cell killing for numerous medical treatments [10,16]. Apart from the traditional photosensitizers, there are a significant number of other photosensitive materials that find applications in the drug delivery system (DDS) as well, for example, polymeric materials (micelles and nanoparticles), porous silica materials, fluorescent quantum dots, liposomes, and hydrogels [14,17]. However, many of these approaches suffer from a general lack of targeting effects and hence are prone to off-target toxicity and limited therapeutic window. 

Therefore, the emerging new concepts, advances in the technology, and the successful cases of improved drug performance and pharmacological activity prompt this review on the light-triggered strategy in drug conjugates. The paper focuses more on photoresponsive linkers in “prodrug”-like approach and antibody–drug conjugates strategy, exploring their implementations for better pharmacokinetics and therapeutic index in the drug discovery.

## 2. Photoresponsive Linker 

In light-triggered drug conjugate, a photoremovable protecting group (PPG) or photocage is used to mask the payload by covalent bond conjugation, making it inactive during circulation. This bond can then be broken by external stimulation through several types of reaction pathways, such as photoisomerization, photo-crosslinking (addition to other molecules), photocleavage, rearrangement reaction, photo enolization, photo-induced phase change, and photo-induced energy conversion [6,14,18,19]. 

Depending on its different applications, there are several criteria for designing a good PPG. In general, it should have a high quantum yield ϕ to promote better clearance in the body regarding in vivo application due to lower PPG requirements. The chromophore should also be benign and has a high absorbance coefficient ϵ at a higher wavelength (above 300 nm) in order to avoid the possible damage to the surrounding biological environment from the absorbance of irradiation of lower wavelength. Moreover, it is preferred for the byproduct to be transparent or inert at this irradiation wavelength in order to avoid competing reactions that may lower the potency of the drug. 

**Figure 2:**
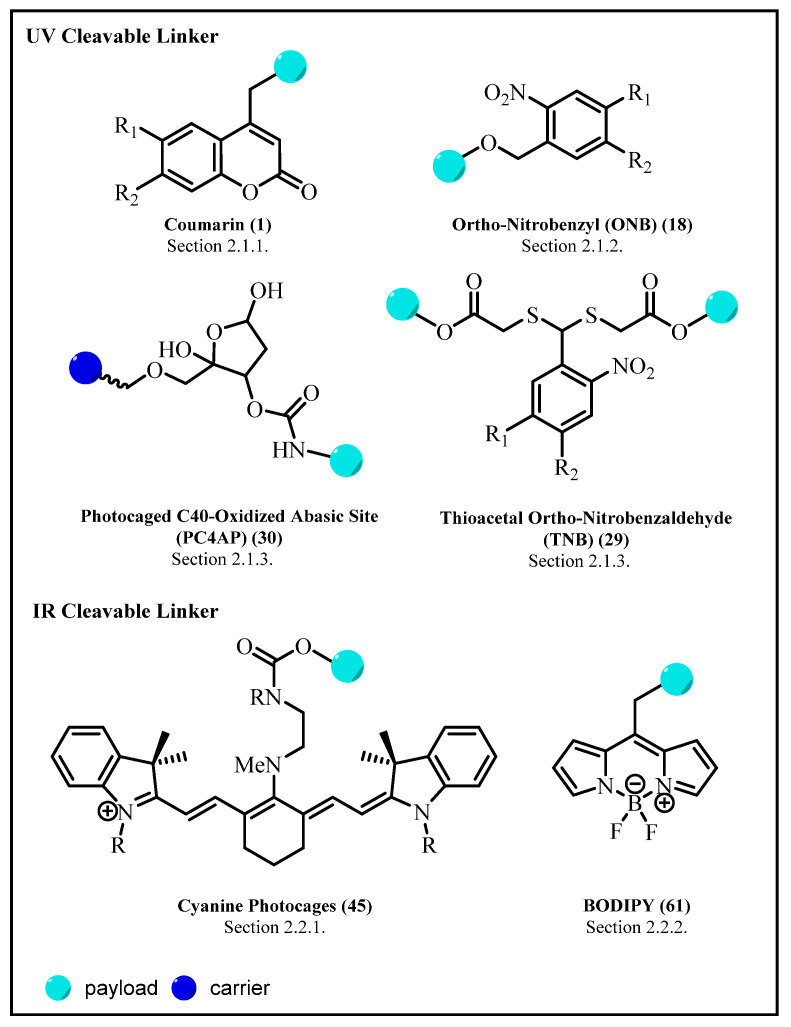
Overview of the structures of UV/Vis and NIR cleavable linkers discussed in this paper.

Additionally, although the production of singlet oxygen to induce cell killing is preferred in PDT, the opposite is required in PPG, and the desired PPG should be biocompatible with the system investigated. In order to obtain an accurate detection response, background activity that may rise from contamination of the deprotected material prior to the irradiation should be avoided. Therefore, the compound should be stable and pure in the media of interest during the sample preparation. As for the protected compound (such as cytotoxic payload), it should be soluble in the targeted biological environment and be able to pass the biological barrier such as cell membranes, and shows binding affinity to the targeted components. Furthermore, the release rate of the cytotoxic payload should exceed the response under investigation. Differentiated by its irradiation wavelength, there are two major approaches to this photoreactive strategy, using an ultraviolet/visible (UV/Vis)-triggered linker and a near-infrared (NIR)-triggered linker; each has its own advantages and disadvantages, which will further be reviewed in the later sections. (For an overview of the protecting groups, see Figure 2).

### 2.1. Ultraviolet/Visible (UV/Vis)-Triggered Linker 

The previous research on light-induced drug delivery systems mainly focused on using irradiation of shorter wavelengths in the ultraviolet spectrum. This is due to its higher energy, enabling to cause disruption to the chemical bond structures, for instance, switching the cis-trans conformation or breaking a covalent bond [14]. Moreover, the relatively simple chemical structures of these UV-triggered PPG make it easier to be synthesized or isolated from other natural resources, lowering the cost of production and facilitating greater availability for application [20]. Among these molecular structures, coumarin and ortho-nitrobenzyl group stand out as the favorite building blocks, along with several other functional motifs.

Nevertheless, this linker strategy also has its own disadvantages, as UV radiation is known to be phototoxic and has low tissue penetration. UV radiation is divided into three sections of the spectrum, each with its distinct biological impact on the living organism: UV-A (λ = 320–400 nm), UV-B (λ = 280–320 nm), and UV-C (λ = 200–280 nm). Studies have shown that single acute exposure to UV irradiation may induce DNA lesions such as pyrimidine dimers and pyrimidine–pyrimidone photoproducts, causing mutation in the DNA. Although DNA has its own repair mechanism, multiple long-term exposures may lead to gradual damage, the photocarcinogenesis. 

Additionally, a high dosage of UV-A irradiation can cause oxidative stress, immunosuppression, and photo aging [14,21]. Apart from phototoxicity, UV irradiation always suffers from low penetration problems as it can only reach up to approximately 100 μm deep in the tissue, hence restricting its usage only for external tissues and the tissues reachable by endoscopic probes [13]. Nevertheless, UV PPG remains to be a group of interesting and important photocleavable linkers for the drug conjugates because of its earlier development and applications, fast release rate, and easier availability. Some processes or methods, for example, optical up-conversion, have been applied in order to overcome the flaws of this UV-triggered linker strategy, which will be elaborated on later in Section 2.1.4.

#### 2.1.1. Coumarin and Its Derivatives

Coumarin is one of the most significant building blocks in medicinal chemistry, thanks to its attractive features such as high bioavailability, low molecular weight, and facile structural modifications. Generally available from natural resources, coumarin structure could be readily constructed via the conventional synthetic methods, such as Knoevenagel or Pechmann condensation, Kostanecki–Robinson, and Reformatsky reaction, to name a few, along with a number of alternative green chemical syntheses that have been reported recently [22,23]. Moreover, coumarin is generally lipophilic, but substituents can be introduced on the aromatic ring to render the coumarin derivatives more lipophilic or hydrophilic [24]. The class of coumarin compounds shows various fascinating pharmacological effects, such as antimicrobial, anticoagulant, anti-inflammatory, neuroprotective, anticonvulsant, antidiabetic, anticorrosive, antiproliferative activities, etc. [24,25,26]. In addition, coumarin derivatives are often used in food and cosmetics due to their aromatic odor. However, excessive or long-term use of this chemical may cause severe adverse effects on the kidney and brain. Hence, the limits of coumarin content in different matrices have been imposed in many countries and institutions [27].

The luminescence properties of coumarin originate from its electron-rich conjugated π–π system, which also contributes to its intrinsic charge transfer properties. Additionally, coumarin has been evaluated and used as the attractive PPG because of its longer absorbance wavelength (up to 500 nm and above at the NIR range) [28] and high photostability due to the conformationally restricted *cis*-cinnamic lactone structure [29]. However, appropriate modifications of coumarin structure with the incorporation of suitable substitutions prove to be essential to increase the intramolecular charge transfer activity and improve the poor fluorescence quantum yield ϕF. Its photophysical properties can be further tuned through the mode of ring fusion, the chemical nature of the extra rings, and electron-donating or electron-withdrawing capacities of substituents [24].

**Figure 3:**
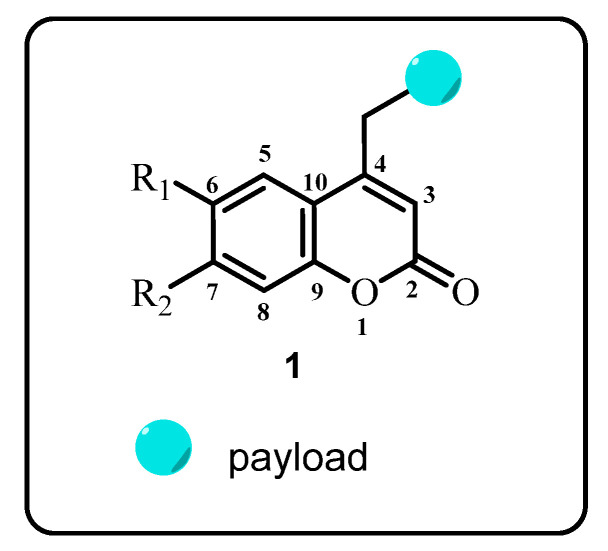
The structures of coumarin derivatives as linker in drug conjugate.

Coumarin-based linker **1** is covalently conjugated on the methylene group at the C4 position with the payload drug. Additionally, there are usually one to two substituents at C6 and C7 positions on the coumarin structure (Figure 3) [30]. The presence of the electron-donating group is prominent to obtain higher quantum yield and good absorbance at a higher wavelength, those photophysical properties of a good photoactive linker used in the drug conjugate. Among coumarin derivatives, 4-hydroxymethyl coumarin derivatives undergo facile photolysis under irradiation [31], making them the most commonly used linker units in drug conjugate.

**Scheme 1:**
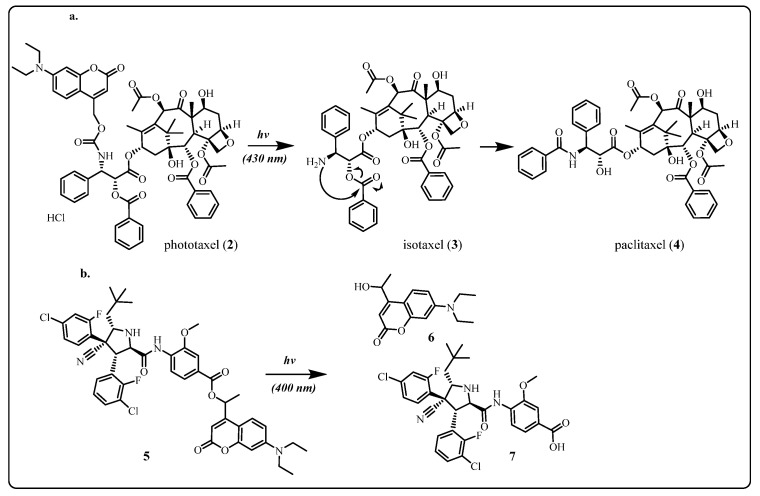
Photoactive release mechanisms of coumarin conjugated prodrugs, phototaxel (2) (**a**) and PPG-Idasanutlin (5) (**b**).

The application potential of coumarin conjugates has been further explored in the “prodrug” approach, where coumarin as photocage helps to improve the pharmacokinetics of certain drugs. Paclitaxel is a potent antimitotic agent that interferes with chromosome isolation and spindle assembly during the G_2_/M stage of cell division [4]. Skwarcynski et al. [32] reported an approach to connect 7-N,N-diethylamino-4-hydroxymethyl coumarin (DCEM) with the amino group of isotaxel (O-acyl isoform of paclitaxel) in order to improve the selectivity and the poor water solubility of paclitaxel. Upon irradiation at the specific wavelength of 430 nm, the photo-cleavage of DCEM from phototaxel (**2**) initiates a spontaneous O-N intramolecular acyl migration, leading to reproduce paclitaxel (**4**) in the target cancer cell (Scheme 1a). The significant results obtained show moderate recovery of the payload with a release yield of 69% after 30 min and no major byproducts detected. 

Another similar approach has been implemented by Hansen et al. in their research effort to promote selective activation of the P53 pathway to the tumor sites [33]. The pro-drug ester (**5**), formed from the conjugation between the coumarin scaffold, 7-(diethylamino)-4-(1-hydroxyethyl)-2H-chromen-2-one (**6**) and idasanutlin (**7**), a MDM2 protein binding agent and P53 activator, is light sensitive and photocleavable upon radiation at 400 nm (Scheme 1b). The good quantum yield, as much as 0.1%, was achieved with selective control of drug release when short 405 nm laser pulses (t pulse, 0.1 s) were employed in the experiment. The irradiation could be applied to individual cancer cells, resulting in selective drug activation in micrometer, a single cell resolution.

Furthermore, the utilization of the coumarin scaffold has evolved into the next generation of the so-called “pro-prodrugs”, where the cytotoxic payload is connected to a photocleavable linker along with another small molecule as a second trigger, aiming to improve the control and selectivity of the drug release. 

**Scheme 2:**
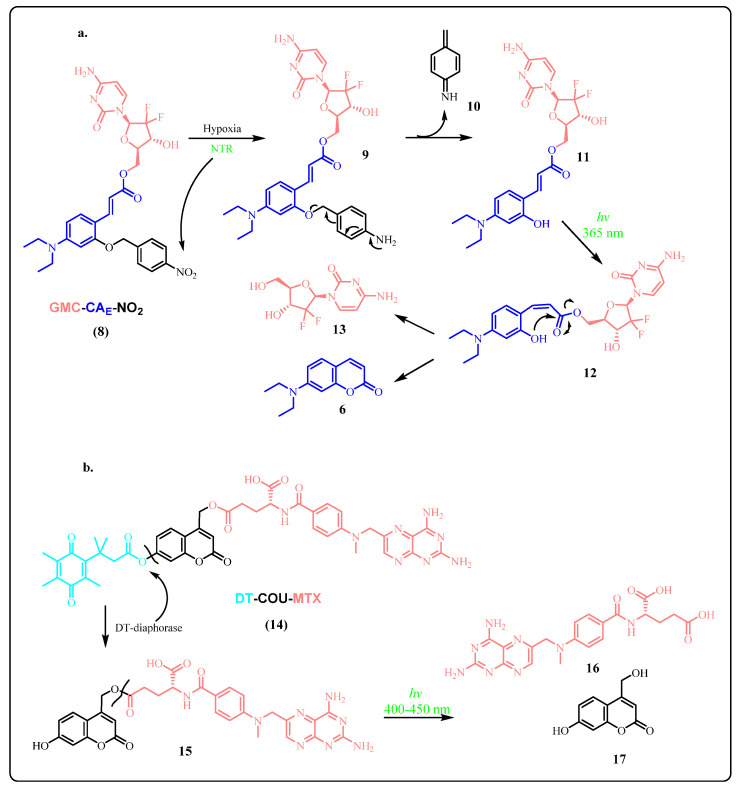
Second trigger-induced release mechanisms of pro-prodrugs, GMC-CA_E_-NO_2_ (**a**) and DT-COU-MTX (**b**).

In this novel approach, the second trigger is a part of the carrier for the drug conjugates and facilitates the target identification. The application has been exemplified by the research performed by Feng et al. [34]. The pro-prodrug (GMC-CA_E_-NO_2_) (**8**), as shown in Scheme 2a, is fabricated by attaching Gemcitabine (GMC) to photoactivated linker *o*-hydroxyl *E*-cinnamic acid (CA_E_) as an ester, which is further modified at the site of the hydroxyl group with the incorporation of the 4-nitrobenzyl group as the hypoxia trigger. The concept takes advantage of the hypoxia condition that often occurs in solid tumor microenvironments and is highly correlated to the overexpression of nitroreductase (NTR). Under normoxic conditions, the nitrobenyl moiety quenches the chromophore, resulting in no activated drug or fluorescent dye being released. However, NTR under the hypoxic condition can selectively catalyze the reduction of NO_2_ of the nitrobenzyl group to amine, and the following 1,6-rearrangement reaction leads to the generation of the GMC-CA_E_ conjugate (**11**) and dienimine byproduct **10**. Upon irradiation at 365 nm, the CA_E_ moiety undergoes photoisomerization from E to Z isomer, rendering the following intramolecular esterification process applicable to successfully release GMC (**13**) and the coumarin dye **6** as the leaving group. 

The second prodrug designed on the same concept is DT-COU-MTX (**14**) (Scheme 2b), developed by Chen et al. [35], containing the anticancer drug methotrexate (MTX), coumarin (COU), and quinone propionic acid (DT), the substrate of DT-diaphorase reductase. Working similarly to GMC-CA_E_-NO_2_, this conjugate prodrug utilizes the overexpression of DT-diaphorase enzyme in the cancerous cell environment as its trigger. The quinone propionic acid, the second trigger, acts as a lock for the photo-induced electron transfer (PET) process of the coumarin PPG. In the presence of DT-diaphorase, the quinone propionic acid moiety is cleaved to release the DT-COU prodrug (**15**), which undergoes another cleavage upon radiation at the range of 400 450 nm to let free of the payload, methotrexate (MTX) (**16**) to exert its biological effect.

More significantly, for both examples above, the prodrugs exhibit long half-life and in vivo stability without significant drug leak in the absence of the two triggers. Additionally, both coumarin molecular fragments from these two prodrugs can also effectively serve as biomarkers for monitoring the drug release rate. In conclusion, the wonderful performance of coumarin in prodrug and pro-prodrug applications, as shown above, demonstrates that coumarin derivatives, with their good photostability and selectivity, are promising and reliable building blocks for the future drug conjugates. The attachment of electron donating groups on C6 and C7 is strongly recommended to achieve high quantum yields, an essential requirement for the successful application of this class of the photoprotecting group.

#### 2.1.2. Ortho-Nitrobenzyl (ONB) Group 

**Scheme 3:**
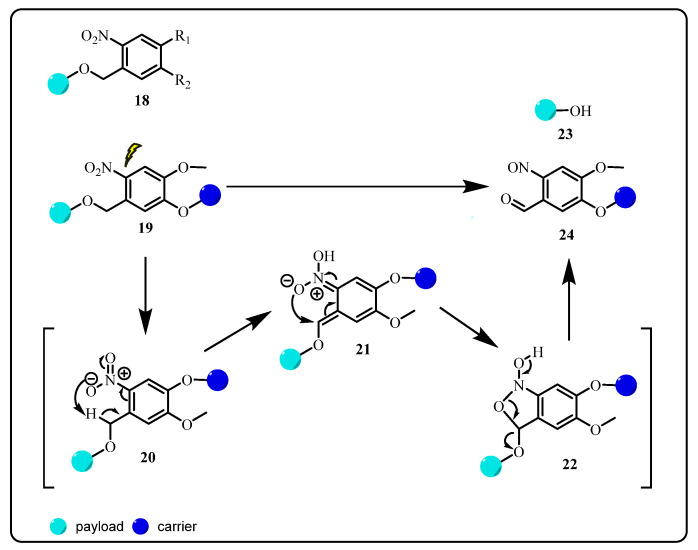
The structure of common ONB building block **18** and its photolysis release mechanism.

The application of the o-nitrobenzyl group (ONB) (**18**) as a photoprotecting group for general use dates back to the 1970s, albeit the reports on its photochemical activities could be traced even prior to that time [36]. The photoactivity of ONB class of PPGs involves a fragmentation mechanism upon irradiation (Scheme 3), in which the intramolecular hydrogen transfers from the benzyl substituent at the ortho position to the nitro group to form the aci-nitro tautomer **21**. The subsequent intramolecular cyclization product **22** undergoes a fragmentation to release the payload **23** as the leaving group and the byproduct of o-nitrosobenzaldehyde **24** [30]. Highly light-absorbent by itself, this molecule competes with the starting drug conjugate for the light absorbance, contributing to the generally low quantum yield of this material (ϕ = 0.01–0.02, compared to other PPGs with ϕ > 0.5), which is an intrinsic disadvantage of ONB as PPG [30,36,37]. Nonetheless, despite a number of alternatives with superior properties available, its versatile modification, highly commercial availability, and straightforward synthesis pathways still make ONB the preferred photocages of choice [37].

In practice, many structural modifications have been attempted to improve the properties of this protecting group. For example, conjugation with more electron drawing groups proves to be very effective, as the incorporation of two methoxy groups on meta (R_1_ (**18**)) and para position (R_2_ (**18**)) to the nitro group can significantly increase the absorbance at higher wavelength and speed up the drug release [38]. At present, ONB linkers find applications in various drug delivery systems, such as nanoparticles drug carriers [39], poly(amidoamine) (PAMAM) dendrimers [40], prodrugs [41,42,43], most recently antibody–drug conjugates [13], etc.

#### 2.1.3. Novel Photo-Triggered Linkers—Thioacetal Ortho-Nitrobenzaldehyde (TNB) Group and Photocaged C40-Oxidized Abasic Site (PC4AP)

**Figure 4:**
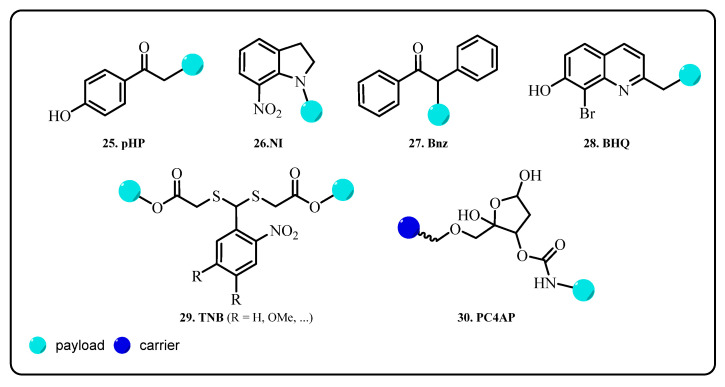
Representative photoprotecting groups used in drug conjugates.

Along with coumarin and the o-nitrobenzyl group as photo-triggered linkers in drug conjugate applications, there has been new emerging interest in developing different alternatives with improved properties and selectivity in order to meet the challenging demands of the drug development. There appear some novel photolabile protecting groups (Figure 4), such as p-hydrophenacyl (pHP) (**25**), nitroindoline (NI) (**26**), benzoin (Bnz) (**27**), 8,7-bromo-7-hydroxylquinoline (BHQ) (**28**), and other structural moieties with their photolysis mechanisms and applications comprehensively studied [38]. Among them, the two most promising groups, the thioacetal ortho-nitrobenzaldehyde (TNB) (**29**) group and photocaged C40-oxidized abasic site (PC4AP) (**30**), are discussed due to their peculiar activation mechanisms and potential in the applications.

##### Photocaged C40-Oxidized Abasic Site (PC4AP)

**Scheme 4:**
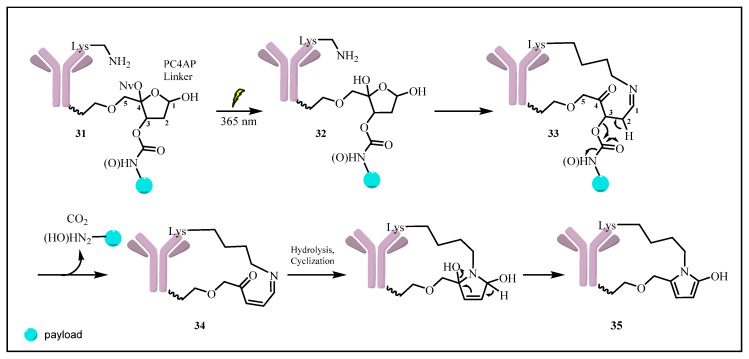
The self-immolation release mechanism of PC4AP antibody–drug conjugate.

In 2019, Zhang et al. [44] reported a novel self-immolated linker, photocaged C40-oxidized abasic site (PC4AP), with its C1-OH and C4-OH being protected by ONB, whereas its C3-OH and C5-OH conjugating to the payload and the carrier respectively. In this drug conjugate **31**, the hydroxyl- or amine- bearing payload is attached to the C3-OH of C4AP via a carbonate or carbamate bond, while the linker is connected to the carrier protein or peptide through an alkyl chain. Upon irradiation at 365 nm, photolysis of OBN liberates the acetal hydroxyl groups on C1 and C4, and the following intramolecular addition reaction of the thus generated acetal **32** occurs with a nearby amine on the carrier to afford the macrocyclic imine intermediate **33**. The subsequent fragmentation reaction leads to cleavage of the carbonate or carbamate moiety and release of the payload along with macrocyclic imine **34** (Scheme 4). The linker remains conjugated to the carrier; after hydrolysis of the imine, intramolecular cyclization produces the macrocyclic heterocyclic species **35**. 

This specially designed linker exhibits one major advantage of the “double trigger release” mechanism as the successful drug release can only happen after 1 h of incubation and in the presence of both light irradiation and primary amines. Trastuzumab-PC4AP-Dox conjugates employed in this research are developed by modification from its acid-labile linker predecessor, trastuzumab-MMCCH-Dox. The results show that the trastuzumab-PC4AP-Dox conjugates have higher stability and faster drug release rate with biocompatible stimulus. Furthermore, the study on SK-BK3 breast cancer cell lines (HER2 positive) shows that the ADC selectively binds to the antigen on SK-BR3 surfaces and has its potency comparable to free doxorubicin (IC_50_ = 203 nM compared to free Dox IC_50_ = 186 nM). Moreover, no cytotoxicity was detected in the absence of a primary amine catalyst inside the cell or without UV irradiation. Featuring high stability and selectivity, as observed above, PC4AP could work as a medium in developing photoresponsive drug conjugates in the future.

##### Thioacetal Ortho-Nitrobenzaldehyde (TNB)

**Figure 5:**
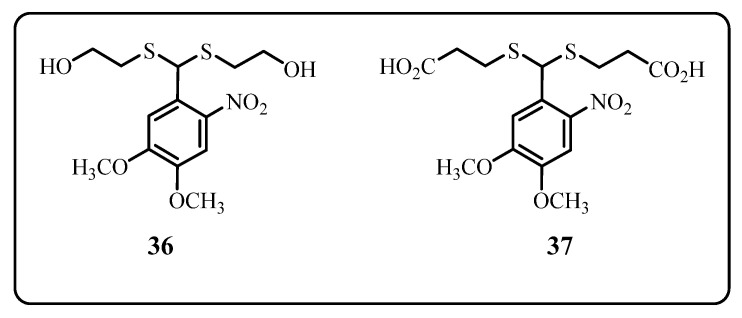
The structures of TNB photocages.

Thioacetal ortho-nitrobenzaldehyde linkers (Figure 5) are interesting and unique photocages to be investigated. Two identical arms with equal symmetry are useful for the conjugation with carrier and payload without causing any regioisomeric issues [45]. TNB linker, designed as a new class of photocleavable linker, has the exclusive dithioacetal structural moiety, similar to ONB, but the ortho-nitrobenzyl carbon is attached to two sulfurs other than one oxygen. The less strong electron-withdrawing effect of sulfur causes a significant bathochromic shift, resulting in stronger absorbance at a longer UV wavelength. Two members of this linker class, TNB(OH) (**36**) and TNB(CO_2_H) (**37**) have similar absorbance profiles with strong absorbance at long UV wavelength (λ_max_ 346 nm, ϵ 4292 M^−1^cm^−1^) and medium UV wavelength (λ_max_ 248 nm, ϵ 11560 M^−1^cm^−1^) respectively. More importantly, TNB linkers are reported to have better quantum yield with ϕ = 0.19–0.24, as compared to ONB linkers with ϕ = 0.01–0.07 [46]. In addition to its unique properties, TNB linkers can be prepared on a large scale in straightforward syntheses from commercially available 6-nitrovertraldehyde [45].

The cleavage mechanism of the thioacetal linker is similar to the one of the ONB linker, as the initial hydrogen shift upon irradiation produces an aci-nitro form. The subsequent nucleophilic addition and other transformations lead to the formation of thioester fragment species bearing payload/linker, which undergo metabolism for further cleavage to release the payload.

Another interesting feature of the TNB linker, as a dual armed photocage, is its capacity to load two conjugates into a single TNB unit and simultaneously release both of them upon radiation of UV light, which renders the TNB conjugate multifunctional to engage in more flexible applications. The strategy is well demonstrated by the drug conjugate taxol-TNB-fluorescein (**38**), which is designed and synthesized along with taxol-TNB-COU and Dox-TNB-COU by Wong et al. in their investigations [45]. Coumarin and fluorescein act as markers for the measurement and real-time monitoring of the drug activation rate.

**Scheme 5:**
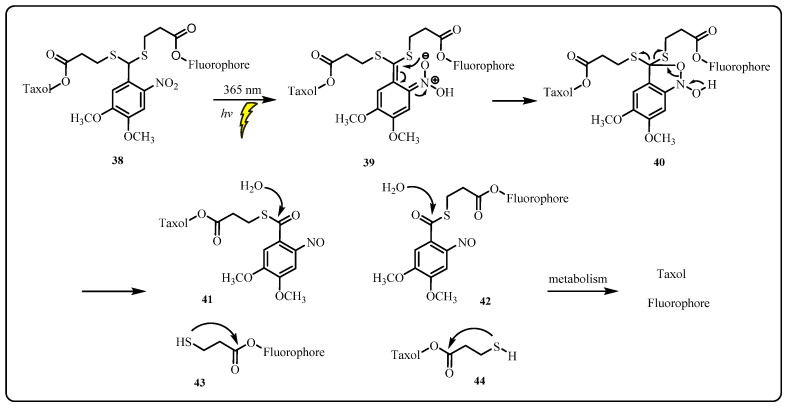
Photocleavage of Taxol-TNB-fluorescein (**38**) via self-immolation pathway.

Irradiation on the conjugate with 365 nm wavelength induces the formation of aci-nitro intermediate, in the meantime initiating the multistep intramolecular self-immolation to produce two pairs of thioesters **41, 42** and mercapto esters **43**, **44** (Scheme 5). The release of taxol and fluorescent reporter can be successfully achieved from the thio esters via transesterification or amidation by nucleophilic biomolecules available in the target environment, or alternatively from intramolecular cyclization reaction on mercapto esters. Satisfactorily, the comparable intracellular cytotoxicity to its free drug form was obtainable in the experiment. 

On the other hand, it is equally important to notice that the TNB linker in the conjugate remains intact without irradiation, and no activity of the payload is detected under a wide range of physiological conditions. However, while exposed to strong oxidants or under extremely acidic conditions, the increased fluorescent activity of the conjugate could be observed, disclosing the vulnerability of the TNB linker as it faces the self-immolation of the weak C-S bond by nucleophilic attack on the ester or carbonate groups. Nonetheless, the overall stability of this class of linker is still within the optimal time limit in therapeutic application. There exists a reliable correlation of the conjugate between the fluorescence activity and the drug release rate, and this dual armed strategy will be a useful monitoring tool for real-time controlled drug release in future investigations. The platform of the TNB linker is also compatible with targeting agents or carriers such as enzymes, antibodies, and dendrimers used in drug conjugation development [46].

#### 2.1.4. The Limitation of UV-Active Linker and the Optional Solutions

Despite the extensive studies on the drug conjugates containing UV-triggered photoresponsive linkers, this strategy still suffers from its fundamental problems, such as UV toxicity, low penetration due to absorbance by intrinsic chromophores, light scattering, etc. The therapeutic window of its application is generally restricted [38]. Some approaches have been made to deal with these limits, mainly by modifying the structure of the linker to incorporate more electron-donating groups or generating more extended π–π conjugation in order to produce a larger bathochromic shift or alternatively using different medical appliances such as endoscopic probes [29].

One of the most promising solutions to these problems is based on optical upconversion processes where low energy pump photons, such as NIR, are converted into high energy output photons, such as UV. Some of the optical upconversion processes which have been comprehensively studied and successfully implemented in light-triggered drug delivery systems (DDSs) include Second Harmonic Generation (SHG), Two-Photon Absorption (TPA), Triplet-triplet Upconversion (TTAUC), and Upconversion Nanoparticles (UCNPs) [14]. Among these processes, TPA and UCNPs are quite often adopted in drug conjugates technology. In principle, TPA requires two photons to be absorbed concurrently by the molecule to reach its excited state and then undergo relaxation through a common pathway. This is usually achieved in practice by using two tightly focused NIR femtosecond lasers to stimulate the molecule into an excitation state, which is then restricted to a (sub-)femtolitre focal volume with small aspect ratios [47].

These processes have seen successful applications in the cleavage of coumarin and ONB linkers of the drug conjugate. UV irradiation at a wavelength of 365 nm is normally used to stimulate the cleavage of ONB linkers, which can be alternatively achieved by TPA two-photon irradiation at 710 nm [48] and 750 nm [47]. Longer wavelength UV is advantageous to be applied to minimize the possible tissue damage by lower UV wavelength in therapeutic application [13]. As for the coumarin linkers, its excitation through single photon absorbance happens at 365 nm [34] and 400–475 nm [32,33,35,49]. It can also go through two-photon excitation at 740 nm [50] and 800 nm [49], albeit showing relatively lower performance compared to the single photon excitation.

Unlike TPA, which requires a high-power pulsed laser to excite the molecule, UCNPs use cheaper and low-power continuous wave (CW) lasers. UCN–PPIX@(Dox)(G5FA), the drug conjugate developed by Wong et al. [46], is the one interesting example of UCNPs, which utilizes the dual arm strategy of TNB linker to conjugate with either folate FAR-targeting (G5) poly(amidoamine) (PAMAM) dendrimer, or doxorubicin payload on one arm, and simultaneously connect with UCN-PPIX (photoporphyrin IX) core on another arm. The incorporation of the UCN structural unit enables this drug conjugate to be cleaved at irradiation up to 980 nm, which does not affect its stability, and thus significantly reduces the toxicity of light irradiation. 

### 2.2. Near-Infrared (NIR), Visible-Light-Triggered Linker Strategy

The intrinsic issues of UV-triggered linkers, such as high phototoxicity and poor penetration, become even more problematic inside human tissue because of the strong scattering and absorbance by water and the presence of other photoresponsive chromophores, such as hemoglobin and melanin [19]; these could not be ultimately resolved even with optical upconversion processes. The recent research has shifted to looking for a new class of linkers that can be activated by irradiation of longer wavelengths. Near-infrared-responsive (NIR) linker drug conjugate offers a promising alternative for an on-demand drug delivery system. 

With its wavelength in the range of 650–900 nm, NIR has the optimum photophysical properties for tissue penetration and can reach up to several centimeters deep due to the reduced interactions between the inside tissue chromophores with the light rays [20]. Additionally, NIR also shows minimal toxicity, and there is no obvious damage to the exposed tissue and blood as compared to UV irradiation. Nonetheless, the less photonic energy of long wavelength NIR also restricts the chemical transformations which are feasible for the IR photocages. Up to now, there are only limited numbers of near-infrared (NIR)-triggered linkers reported in the applications, in which either isomerization or bond cleavage reactions are responsible for the structure breakdown upon irradiation [20]. 

Additionally, the more complex structure of this class of linker, which requires considerable efforts for syntheses, is also related to the problem of self-aggregation, albeit there have already been some attempts to tackle this issue by modifying the fluorophore to be more hydrophilic with the introduction of sulfonate groups [51]. To counteract the low photonic energy of NIR, approaches using two-photon excitations (NIR to UV, TPA upconversion) as discussed in Section 2.1.4 can also be considered, although its efficiency might need to be improved for the optimization of practical applications. 

Cyanine photocages, red-shifted coumarin, BODIPYs, and xanthenes are the representative photocages that have been developed and successfully used in various conjugates [51]. The structures, cleavage mechanisms, and performances of cyanine and BODIPY, the two widely used fluorophores, will be reviewed in detail in the following section.

#### 2.2.1. Cyanine Photocages

**Scheme 6:**
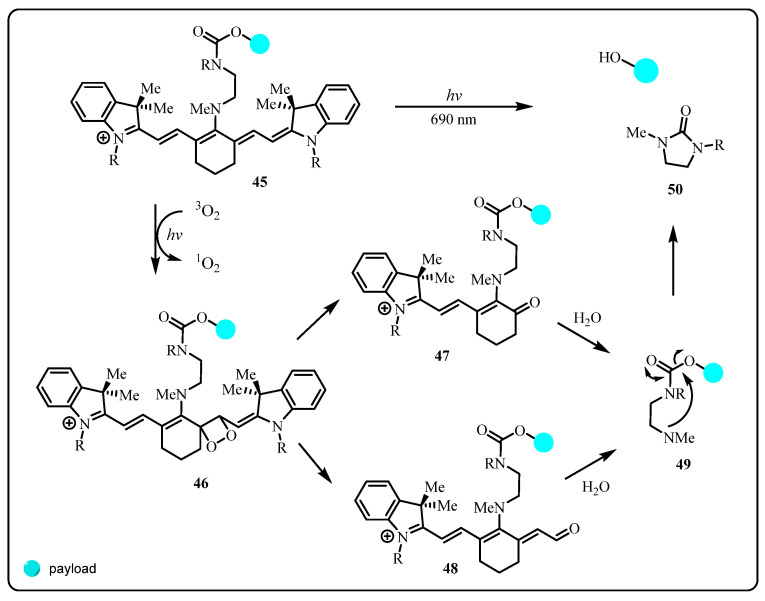
^1^O_2_-mediated oxidative photocleavage of heptamethine cyanine scaffold.

Heptamethine cyanine fluorophore scaffold (**45**) (Scheme 6) has excellent fluorescent properties, which enables easy control of the drug release from the conjugate and also tracking of the process with optical imaging technology [51]. Its drug release mechanism relies on the oxidative addition by self-sensitized singlet oxygen (^1^O_2_). The thermally labile dioxetane intermediates such as **46** from this addition are subjected to the bond cleavage at different positions, resulting in the formation of carbonyl compounds **47** or **48,** respectively, containing the hydrolytically labile enamine structure C=C-N after these transformations [52]. The free secondary amine **49** from the hydrolysis participates in a cyclization reaction to finally release the payloads. The half-life release time for the conjugates via the multiple-step cleavage sequence upon irradiation (30 J·cm^−2^ in vitro, to 100 J·cm^−2^ in vivo) is about 20–30 min, which is unfavorable for some biological processes that require high temporal resolution, and the passive diffusion of partially uncaged intermediates may disrupt spatial patterning under the physiological pH conditions.

However, the application of this fluorophore becomes compatible with various targeting agents, especially monoclonal antibodies (mAb) [53]. One example of its successful clinical applications is the use of cyanine dye IR800-CW conjugated to monoclonal antibodies in various surgical procedures for solid tumors [54,55,56]. Results obtained from the research show that the drug has minimum toxicity and is well tolerated in the body. 

**Scheme 7:**
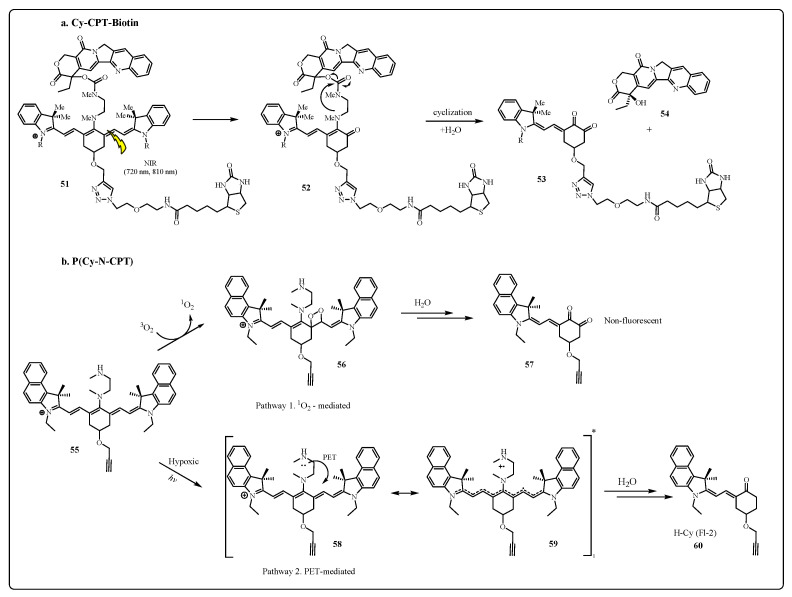
(**a**) Photolysis release mechanism of Cy-CPT-Biotin conjugate; (**b**) Competitive photoactivation pathways for photocage P(Cy-N-CPT) (**55**).

This fluorophore dye was also used in Cy-CPT-Biotin, another photocaged prodrug **51** developed by Guo et al. (Scheme 7a) [57]. Dialkylamine-substituted tricarboncyanine linkers are covalently connected to biotin (Vitamin B7) along with comptothecin (CPT) payload via a carbamate bond. The targeting agent is incorporated through a triazole linkage, a structural moiety constructed by click reaction. Very impressively, the distribution of the prodrug during circulation could be monitored and tracked as the conjugate is exposed to an 810 nm ray of light. Furthermore, photochemical bond cleavage occurs to form the active intermediate **52** upon irradiation at 535 nm, and the subsequent intramolecular cyclization releases comptothecin (**54**) payload and byproduct **53**.

Nonetheless, the photolysis reliance of cyanine fluorophore scaffold on the concentration of singlet oxygen in the tumor tissue is the major factor that hinders the application of this type of linker under hypoxia conditions, which is more often the case in many tumor tissues. Recently, photocage P(Cy-N-CPT) (**55**), a hypoxia suitable dialkylamine-substituted cyanine (Cy-NH) linker, was developed by Zhang et al. [58]. This diblock copolymer can be activated under acidic conditions in a hypoxic tumor environment and undergoes the subsequent photolysis for cleavage. Upon irradiation with NIR light of wavelength > 650 nm, the photo-induced electron transfer (PET) process under hypoxia occurs from the amino group to the methine chain of Cy-NH, and the retro-aldol reaction and hydrolysis of the excited state **58** and intermediate **59** proceed to release the diamine, the functional fragment for payload attachment (Scheme 7b). It was reported that the fluorophore shows similar absorbance spectral change under both normoxic and hypoxia conditions. Additionally, these dual-mode optical signals also allow more precise remote spatiotemporal control in a hypoxic environment.

Along with its low toxicity and perfect compatibility with the targeting agent, structural modifications of this cationic fluorophore to introduce functional groups such as sulfonates can tune its water solubility and effectively control the aggregation of its biomolecule conjugate [59]. Furthermore, this photocaged molecule features a straightforward attachment to various targeting agents. Its interesting application in the development of antibody–drug conjugates (ADC) will be elaborated on in Section 3.2.

#### 2.2.2. BODIPY

**Figure 6:**
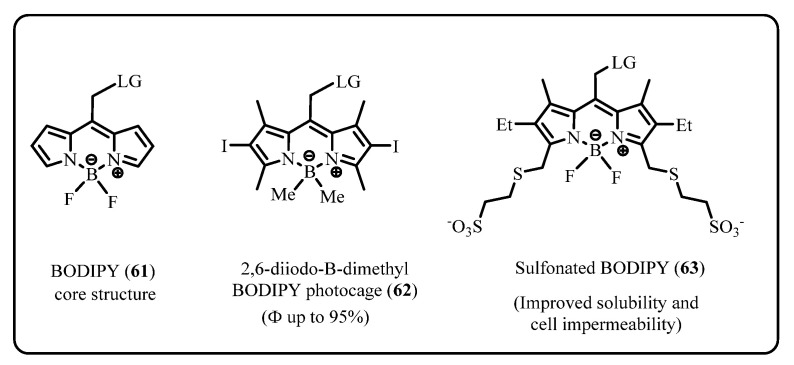
The structures of BODIPY (**61**) and its functionalized derivatives (**62**, **63**).

BODIPY (**61**) (Figure 6) and its derivatives have gained more attention in recent years due to their biological characteristics and ideal photophysical properties for photocaging. Biocompatible with a wide range of materials, they also have a narrow excitation band and can be activated at wavelengths up to 700 nm, with the strongest absorbance at the green spectrum (λ ≈ 520 nm). Their structures are adaptable, and various chemical modifications may result in a bathochromic shift with an improvement in quantum efficiency [60]. For example, one of the BODIPY derivatives, 2,6-diiodo-B-dimethyl BODIPY photocage (**62**), as reported by Slanina et al., can have its quantum yield improved up to 95% in the release of chloride, a mediocre leaving group.

**Scheme 8:**
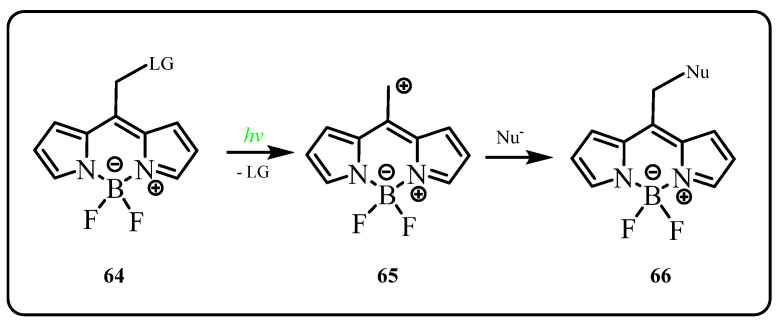
Photochemical S_N_1 reaction, the cleavage mechanism of BODIPYs.

For the class of BODIPY photocage, such as **64**, the general release mechanism involves a photochemical S_N_1 reaction. Upon irradiation, the fragile allylic bond between the BODIPY core and its leaving group breaks to produce the carbocation intermediate (**65**) for a further nucleophilic attack from solvent, and the leaving group or the payload in the potential medicinal applications [61] (Scheme 8). Another interesting feature of the BODIPY-N-NO hybrid is to generate the radical oxygen along with NO upon irradiation, which is capable of inducing cell death [52].

The extremely hydrophobic BODIPY is suitable to be used in the investigations on intracellular interactions but not for extracellular targeting receptors in the plasma membrane. Furthermore, the less functionalized structure makes it difficult to link with other important bioconjugates and restricts its further biological applications. The physical and chemical characteristics of this molecule have been greatly improved by a remote sulfonation reaction developed by Kand et al. to introduce the sulfonate functional group at two and six positions. The disulfonated BODIPY (**63**) from this structural modification, with improved water solubility and cell impermeability, has been used for modulation of cell-surface receptors and localization control by visible-light photoactivation of signaling molecules [62]. Thus far, BODIPY derivatives have not seen many successful applications as visible-light photocaging in drug conjugates; however, its biocompatibility along with good quantum yield makes this structure a promising potential linker to be developed.

### 2.3. Diazido Platinum(IV) Complexes for Photoactivated Anticancer Chemotherapy(PACT)

**Figure 7:**
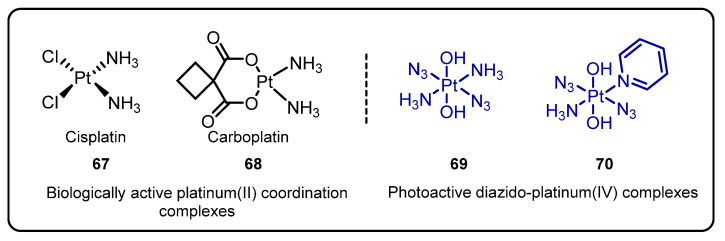
Some of the platinum complexes developed as anticancer drugs.

The discovery of cisplatin (**67**) in 1968 by Rosenberg heralded a new era for platinum-based anticancer drugs, which are now used by over 40% of all cancer patients in chemotherapy. Cisplatin has a mechanism of action similar to that of alkylating agents and forms covalent adducts with DNA, showing its cytotoxic activity as antiproliferative [63,64,65,66]. The active coordination complexes, such as cisplatin, carboplatin (**68**), and other derivatives, all feature platinum in the 2+ oxidation state and almost invariably adopt the square-planar coordination geometry [67,68,69] (Figure 7). On the contrary, the biological activities of platinum(IV) complexes (**69**, **70**), which have a d^6^ electronic configuration and are more kinetically inert to ligand substitution and more stable under physiological conditions, are believed to be originally from Pt(II) complexes, the reduced species form Pt(IV) by bioreductants (e.g., GSH, ascorbic acid, cysteine as shown in the earlier experiments) [70,71,72].

The photoreductive Pt(IV) complexes with azide ligands perfectly incorporate the photodecomposition of Pt(IV) complex with the light sensitivity of azido complexes, making itself the potential PACT (photoactivated anticancer chemotherapy) prodrugs. Since the first report of such a complex with a structure of *trans*-[Pt(N_3_)_2_(CN)_4_]^2−^ by Vogler et al. in 1978, prominent progress has been achieved in the development of complexes of new structures, understanding the mechanisms of the activation and phototoxicties in medicinal application [73,74,75,76,77].

The physical and chemical properties of Pt(IV) complexes ensure that these prodrugs can reach cancer cells after administration. On irradiation, they can be selectively reduced/activated to Pt(II) complexes and exert their cytotoxicity on site. On the other hand, it is essential to maintain the higher dark stability and resistance to bio-reductants in order to decrease off-target toxicity. The structural significances, which greatly affect the potencies of the Pt(IV) complexes, include *trans* geometry configuration, the selections of the non-leaving ligand, and the axial ligands [78,79,80]. Axial ligands, predominately by hydroxide and ester, can be utilized to improve the properties of the complex with better selectivity and cytotoxicity and also extend the conjugate for other applications. Non-leaving ligands are mostly various amine functional moieties, which bestow the stability of the complex and provide the active pharmacophore structure. The leaving ligands, released upon irradiation, affect the reduction potential and play key roles in the activation of Pt(IV) complexes. Diazido-Pt(IV) complexes with the general formula [Pt(N_3_)_2_(L)(L’)(OR)(OR’)] become the photoactive prodrugs of choice in the developing process because of the promising prospects, such as excellent dark stability and photobiological properties.

**Figure 8:**
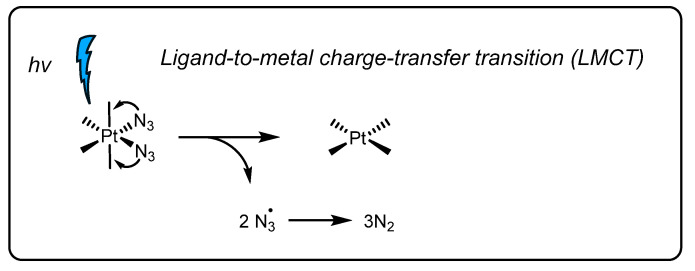
A plausible mechanism for the photoreduction of diazido-Pt(IV) complexes.

Photodecomposition of diazido Pt(IV) complexes is triggered upon irradiation, as ligand-to-metal (N_3_→Pt) charge-transfer transition (LMCT) results in the release of two azide ligands as N_3_•radicals which can combine to form N_2_ molecules and the photoreduction to afford anticancer active Pt(II) species. (Figure 8) [81,82,83]. The diazido Pt(IV) complexes, which can be activated at a longer wavelength (>300 nm), generally feature *trans* configuration, bidentate chelating ligands, non-leaving ligands with steric properties, and electron-donating activity such as the π-acceptor pyridine ligand due to the feasible red-shifts of LMCT band. 

In recent years, the development of a new generation of diazido Pt(IV) complexes as anticancer prodrugs focuses on the modifications of axial ligands. The strategy proved to be effective in improving the selectivity and cytotoxicity of the complex and has also been used to conjugate with anticancer drugs or cancer targeting vectors in order to produce multi-action prodrugs for the photoactivated chemotherapy [72,84,85,86]. Improved potencies from synergistic effects are achieved from the combination of different cellular targets and action mechanisms.

In 2021, Sadler et al. reported the coumarin derivative of *trans*-dihydroxido Pt(IV) complex **71**, which exhibits increased photocytotoxicity in cancer cell lines. Coumarin itself can act as a light-harvesting antenna and also possesses intrinsic anticancer activity [74]. Very interestingly, the in-cell behavior of these prodrugs could be investigated and visualized by using synchrotron techniques, showing the changes in cellular morphology and Pt localization upon treatment with and without light irradiation.

**Figure 9:**
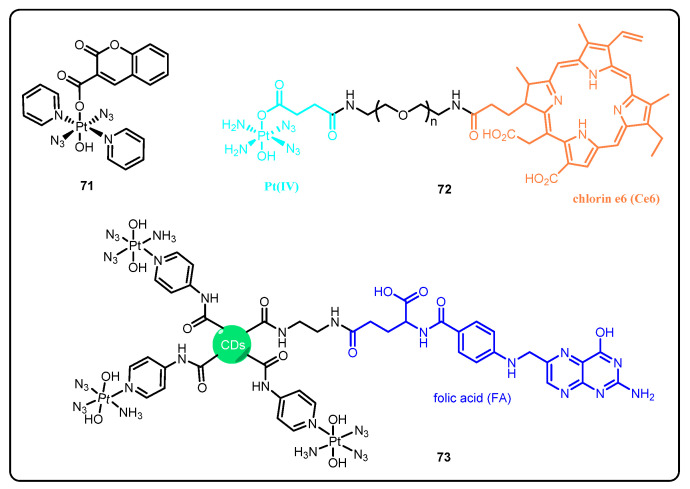
Diazido platinum(IV) complexes developed as anticancer prodrugs.

Oxygen and Pt(II) self-generating multifunctional nanocomposites were designed to overcome the hypoxia-triggered PDT resistance. The amphiphilic oligomer Ce6-PEG-Pt(IV) (**72**) can self-assemble into micelles and then co-assembles with NaYbF4: Tm@CaF_2_ to make upconversion nanoparticles (UCNPs) embedded nanoparticles (Figure 9). The irradiation with a 980 nm laser triggers the generation of O_2_ for consumption in the PDT and also releases active Pt(II) for synergistic photo-chemo therapy [87].

A novel Pt(IV)–azide@carbon dots (CDs) (**73**) for controlled delivery of diazido-Pt(IV) complexes was reported by Liu et al. The conjugated folic acid as targeting vectors helps to improve the selectivity and enables the accumulation of the complex in the folate-receptor overexpressed cancer cells [88]. Upon irradiation with UV or visible light, a similar level of therapeutic efficacy is achieved as that of cisplatin. 

Diazido-Pt(IV) complexes with good cytotoxic activity and high dark stability make a special class of PACT anticancer drugs. Their targeting ability and pharmacological properties could be easily modulated by the further functionalization of the complex, mainly the modification of axial ligands. The success of developing platinum anticancer prodrugs for in vivo testing or clinical trials very much relies on the optimization of the physical, chemical, and biological properties of these complexes, such as good aqueous solubility, targeted drug delivery with improved accumulation, longer wavelength activation for deeper tissue penetration, rapid activation for spatial and temporal control and high photocytotoxicity, which still remains to be challenging but also is becoming promising as so many efforts are ongoing now.

## 3. Application in Antibody–Drug Conjugate (ADC)

### 3.1. ADC in Cancer Therapy

Among the studies of drug conjugates and cancer therapeutics, the use of antibodies has been attracting widespread interest due to its active targeting activity along with ready availability and cost-effective production [89]. Monoclonal antibody (mAb), as the targeting agent, offers several distinct advantages compared to other conventional chemotherapeutic agents, such as great selectivity and long half-life [1]. Its application dates back to 1997, when the first mAb, rituximab, attained approval. Since then, there are currently more than 20 approved monoclonal antibodies available for various therapeutic use [90].

One of the most promising applications, antibody–drug conjugates (ADC), the original concept of the magic bullet, has already evolved through three generations, with 7 ADCs approved in the market, and around 70 ADCs are on clinical trial [13,91,92]. The first generation of ADC utilizes acid-labile linkers such as carbonate and hydrazone bonds. The examples of this generation ADC in the market are Gemtuzumab Ozogamicin (Mylotarg^®^) and Inotuzumab Ozogamicin (Besponsa^®^) [93]. However, Mylotarg was withdrawn from the market in 2010, mainly due to the instability of hydrazone linkers, which offers no significant benefits to outweigh the other toxic effects such as infusion reactions, pulmonary and hepatoxicity [91]. Mylotarg also suffered from its low chemical, manufacturing, and control (CMC) properties, as well as low drug to antibody ratio (DAR). Other first-generation ADC, such as BR96-doxorubicin and KS1/4-methotrexate utilizing non-cleavable linker, did not show promising data, with potency lower than their free drug counterparts [1,91].

More stable linkers were widely adopted in the development of the second-generation ADC, such as cathepsin cleavable valine-citrulline (Val-Cit) linker in brentuximab vedotin (Adcetris^®^) and non-cleavable thioester linker in ado-trastuzumab emtansine (Kadcyla^®^). Apart from the improved linker stabilities, more potent microtubule-targeting agents (such as auristatin and maytansinoids derivatives) were used as the cytotoxic payload to improve the general potency of ADC [1].

Nonetheless, the second-generation ADC still utilizes non-specific site conjugation for the incorporation of antibodies, which generally produces ADC as heterogeneous mixtures with the drug to antibody ratio (DAR) varying from zero to eight. Furthermore, lower potency could be one of the problems due to competition from those unconjugated antibodies available in the product [90]. Additionally, off-target toxicity is still prevalent due to the non-differentiated triggers present in healthy tissue; for instance, the Val-Cit linker becomes unstable in the presence of carboxylesterase 1C (Ces 1C) enzyme, as reported in recent research on mouse models [7]. Similar to second generation ADC, the third generation ADC predominantly uses enzyme cleavable linker, albeit applying it in side-specific conjugation to the antibody to improve the pharmacokinetics as well as its DAR [94].

Although with continuous and extensive efforts, the conventional ADC with internal trigger-linker is still plagued with relatively poor therapeutic index, which has limited the implementations in the pharmaceutical industry, where there were 23 out of 55 conventional ADCs that were terminated due to this very same reason [95]. There have been emerging interests in the use of external stimuli, especially light irradiation, as the new trigger for the ADC linker. ADC with good stability and photo-controlled release seems to be an attractive and practical solution for future cancer therapy.

### 3.2. Application of Photoresponsive Linker in ADC

Many factors have to be taken into consideration for a successful photoresponsive ADC, which integrates a photocleavable structure into the linker, exhibits good stability under the physiologic conditions, and can rapidly release free cytotoxins upon irradiation in the meantime maintaining a targeting effect similar to that of the naked antibody and exerting the essential cytotoxicity for biological significance. The development is still in its infant phase but has been growing slowly and steadily. One of the two most recent and prominent contributions was from Li’s group utilizing UV cleavable ONB linker to modify and improve the stability of Val-Cit linker in ADC, and another one from Nani’s group with IR cleavable cyanine photocage for its biocompatibility and relatively safe nature of IR light.

**Scheme 9:**
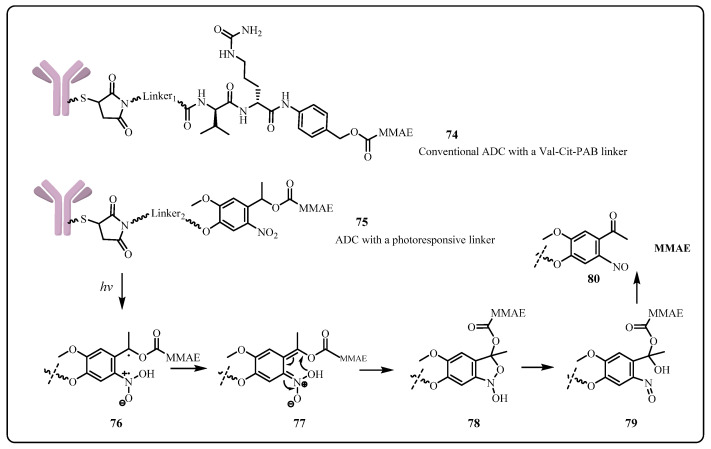
Different release pathways of conventional ADC and ADC with photoactive linker.

The novel ADC, developed by Li, et al. in 2021 [13], contains potent monomethyl auristatin E (MMAE) as its payload and antibody mil40 (CTR20180362), a new antibody currently in the clinical research stage with biosimilarity to transtuzumab. As reported, the valine-citruline (Val-Cit) linker was modified in the research by replacing para-aminobenzyl (PAB) moiety (**74**) with 4,5-dimethoxy-2-nitrobenzyl derivatives (DMBD) (**75**), in order to reduce the off-target toxicity due to some unspecific cleavage. Upon radiation at 365 nm, the nitrobenzyl group of linkers absorbs a photon and enters an excited triplet state; hydrogen abstraction leads to a resonance-stabilized benzylic diradical **76**, which undergoes a rearrangement to generate aci-nitro species **77**. Collapsing of the unstable five-membered cyclic acetal ester **78** leads to the liberated payload with nitrobenzaldehyde as the byproduct of the reaction sequence (Scheme 9).

The drug satisfactorily exhibits comparable performance to the current ADCs in the market with a long half-life (similar to unconjugated mil40) and good plasma stability (t_1/2_ > 6 days under normal light with only <1% MMAE released), along with a significant tumor-targeting effect in vivo. This significant tumor-targeting activity, which can be seen as the ADC undergoing rapid payload release upon radiation (EC_50_ = 0.04 nmol·L^−1^), is higher than unirradiated ADC by 50 times and reaches its plateau after 10 min of irradiation. Nevertheless, despite the good targeting effects and stability for its promising therapeutic application, further investigation and improvement of this ADC are ongoing as in vivo Cys-linker-degradation was reported in a recent study. 

**Figure 10:**
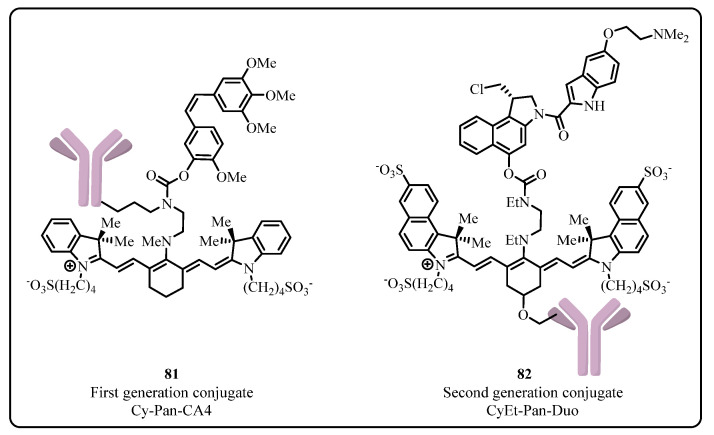
First and second generations of ADC containing cyanine photocage linkers.

The biocompatibility of cyanine photocages with monoclonal antibodies, as discussed in Section 2.2.1, makes it an excellent building block for ADC. In 2015, Nani et al. reported the first generation of cyanine type ADC **81**, using microtubule polymerization inhibitor, Combretastatin A4 (CA4) as its payload, and panintumumab monoclonal antibody as its targeting agent (Figure 10). The conjugate shows significant tumor uptake, along with good stability in vivo and minimal release of payload without irradiation in human plasma (<1% after 72 h at 37 °C). On the other hand, the potential of this conjugate is relatively limited by the minimal tumor shrinkage due to the less potency of its CA4 payload and the prevalent self-aggregation problem in bioconjugations. These drawbacks were later addressed in 2017 by the same authors using a more potent DNA alkylator agent, Duocarmycin (IC_50_ ≈ 20–50 pM), as its payload and modifying the Cyanine scaffold core structure with the addition of sulfonate benz[e] indole ring and alkyne handle to facilitate better bioconjugation (**82**, Figure 10). These alterations led to improved stabilization and less self-aggregation, thus increasing the therapeutic index by up to 600 times and more significant tumor shrinkage. Additionally, a bathochromic shift of the absorbance maxima can also be observed as the linker cleavage occurs at λ = 690 nm and λ = 780 nm. 

Light-controlled linker with its internal stability and good pharmacokinetic properties exhibits a great application potential in the development of future ADC, as reviewed above. The irradiation activation can be conveniently achieved by using fiber optic or endoscopic probes to transmit the required light to the site of action or directly by irradiating the lesion during surgery. This external trigger mechanism provides a favorable option to circumvent the intrinsic problems of the conventional conjugate, such as off-target toxicity, poor therapeutic index, etc. In the meantime, it allows more possible manipulations in the development to improve the potency and pharmacokinetic properties. Further investigations are going to focus on the optimization of the photocleavable linker structures and developing a better understanding of the mechanism of ADCs entering the cells, which will witness the important progress in the therapeutic treatment of diseases. 

### 3.3. Photoimmunotherapy (PIT)

Monoclonal antibodies, targeting surface antigens expressed on tumor cells, are widely used in antibody-based molecular therapy to minimize the side effects. The recent advances in molecular and protein engineering technologies make it accessible to select more efficient therapeutic mAbs with increased affinity and specificity, efficient cell distribution, and lower immunogenicity, which stimulate further biological investigations and clinical applications. The conventional immunotherapies activate the adaptive immune effectors, such as T cells, to induce antitumor activity, but do not directly destroy cancer cells. In contrast, Near-infrared photoimmunotherapy (NIR-PIT), conjugating a monoclonal antibody to a photoabsorbing dye, IRDye700DX (IR700), has demonstrated its efficiency as a theranostic cancer strategy and for highly selective treatment [96,97,98].

**Scheme 10:**
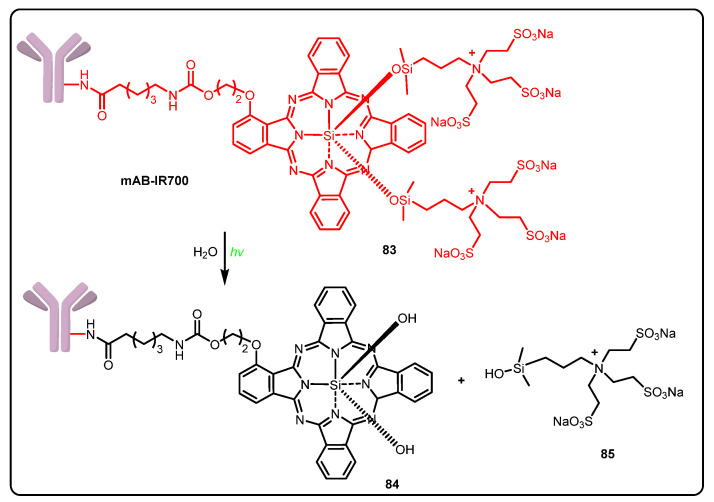
A schematic for the photochemical reaction of NIR-PIT.

Administration of the antibody–photoabsorber conjugate (APC) via intravenous injection leads to the accumulation of the conjugates around the specific cancer cells to which the antibody is attached. Upon irradiation by near-infrared (NIR) light, releases of hydrophilic side chains **85** of IR700 via photochemical ligand reaction render the complex molecule to become extremely hydrophobic (Scheme 10). The enhanced permeability and retention (EPR) in the tumor vessels and the impaired cellular membrane function by aggregation and solubility changes might cause immediate necrosis. On the other hand, the targeted cell death can be induced by immunogenic actions such as rapid maturation of dendritic cells and priming of cancer-specific cytotoxic T cells from cancer-specific antigens, which are released into the tumor microenvironment on photoactivation of NIR-PIT [99,100,101]. 

NIR-PIT selectively destroys cancer cells, leading to immunogenic cell death that initiates special local features of near-infrared laser light used in the therapy, such as good tissue penetration, nonthermal and not ionizing radiation; thus, there is no damage to cellular DNA, making NIR-PIT selective for the treatment of superficial tumors and tumors in the lung and pleural cavity with good control of off-target effects. The efficacy could be further improved by increasing the frequency of administration of APCs and the repeated NIR light treatment for the residual or recurrent disease. The continuous efforts and explorations have seen the clinical approval in Japan for NIR-PIT in recurrent head and neck squamous cell cancer (HNSCC) treatment, which is reaching phase III clinical trial now. The combinations with immune-checkpoint inhibitors to target immune suppressive cells in the tumor microenvironment will likely extend the potential of this strategy and forebode more promising applications [102,103,104,105].

## 4. Conclusions

Despite the significant development of drugs along with the novel treatment methods available in clinical cancer therapy, the prevalence of cancer still remains to be a formidable challenge, and huge obstacles are there to be solved. The increasing risk of toxicity on patients and limited therapeutic window are the major problems in the current chemotherapies. Various drug delivery systems have been developed to improve the pharmacokinetic and pharmacodynamic properties of these drugs in order to effectively address these issues.

Drug conjugate or prodrug is one of the most successful approaches for these strategies, using various linkers to mask the activity of the drug and connect with drug carrier and targeting agents, which effectively increase the selectivity of the drugs and, very importantly, improve the ADME properties to subtly circumvent all sorts of difficulties of the complex biological system to deliver the potent payload at the right place and at the right time. Linker, as its name implies, is the structural hub for assembly of the other essential components for becoming a complete pro-drug and also plays a significant role in practice to release the payload. 

In recent years, photocleavable linkers have been developed as a non-invasive technology, allowing the controlled release of the active substance with high spatiotemporal precision. Along with the utilization of carriers that take advantage of active targeting strategies such as monoclonal antibodies, this strategy of drug delivery becomes a powerful tool in clinical cancer therapy. As seen from the research on the photoremovable linkers so far, the drug conjugates, especially ADC, have shown minimal off-target toxicity without irradiation and increased stability compared to other conventional ADCs utilizing an internal trigger linker strategy. In addition, the linker modulates ADC stability in the systemic circulation and payload release efficiency, in the meantime affecting ADC pharmacokinetic (PK), efficacy and toxicity profiles. Conjugation chemistry, linker length, and linker steric hindrance are among the key linker parameters to be considered in order to achieve a balance between stability and efficacy in the integrated strategies.

This orthogonal release approach, which has demonstrated its potential from previous applications, will be further explored in future research. The discovery of a new release mechanism and novel structure will benefit the development of new photoresponsive linkers, which can be applicable for anticancer drug conjugates and other delivery applications—in particular, those that require non-invasive or spatiotemporal drug/probe activation. The external tools applied to actively trigger the drug release can be extended from the current light irradiation to other energy forms, such as ultrasound. The high spatial and temporal resolution of ultrasound enables precise location, targeting, and timing of drug delivery and tissue sensitization, which could revolutionize the pharmaceutical approaches forever.

## Data Availability

Data sharing not applicable.
